# Anti-aging effect of Hedgehog signaling

**DOI:** 10.1038/s12276-025-01626-7

**Published:** 2026-02-12

**Authors:** Ji-Hoon Kim, Jae Yoon Hwang, Ji Hye Jun, Anna Mae Diehl

**Affiliations:** 1 Department of Pathology, Duke University, Durham, NC USA; 2 Division of Life Science, College of Natural Sciences, Gyeongsang National University, Jinju, Republic of Korea; 3 Division of Gastroenterology, Department of Medicine, Duke University, Durham, NC USA

**Keywords:** Senescence, Biological therapy

## Abstract

Aging is characterized by the progressive loss of physiological integrity, leading to impaired tissue function and increased vulnerability to chronic diseases. Although the Hedgehog (Hh) signaling pathway is well established as a key regulator of embryonic development and tumorigenesis, emerging evidence suggests it also plays vital roles in adult tissue maintenance, regeneration and immune modulation—processes that are intimately linked to aging. Here we synthesize recent findings demonstrating that the controlled activation of Hh signaling across diverse tissues, including the brain, liver, heart, lung, bone, skin and adipose tissue, can counteract hallmark features of aging such as stem cell exhaustion, mitochondrial dysfunction and chronic inflammation. In preclinical models, Hh pathway modulation enhances tissue regeneration, supports progenitor cell function and suppresses senescence-associated secretory phenotypes. Promising therapeutic strategies—ranging from gene delivery to pharmacological agonists—have shown efficacy in mitigating age-related decline, though challenges remain regarding tissue specificity, long-term safety and tumorigenic risk. By integrating insights from developmental biology, regenerative medicine and geroscience, this Review positions Hh signaling as a compelling target for anti-aging interventions aimed at preserving organ function and extending healthspan.

## Hedgehog signaling enhances tissue regeneration in aging

Aging weakens the body’s ability to maintain balance and repair damage, increasing vulnerability to disease. This study reveals that the Hedgehog (Hh) signaling pathway plays a crucial role in preserving tissue integrity and regenerative capacity. Using animal models, researchers found that activating Hh signaling in multiple tissues such as the liver and brain enhances tissue repair and mitigates age-related functional decline. These findings suggest that targeting Hh signaling could be a promising strategy to promote healthy aging by enhancing regeneration and alleviating age-related dysfunction.

This summary was initially drafted using artificial intelligence, then revised and fact-checked by the author.

## Introduction

**Fig. 1:**
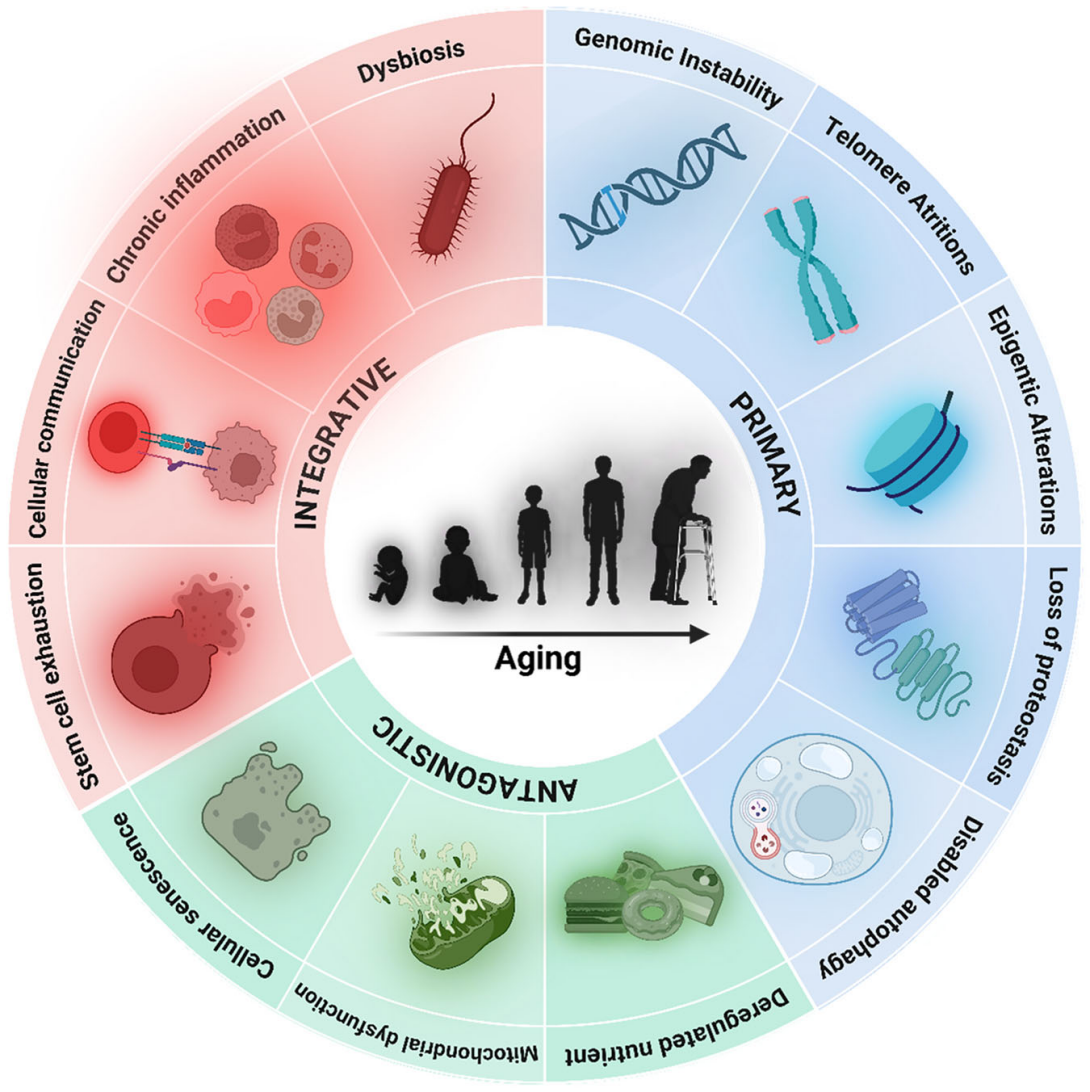
Conceptual framework: hallmarks of aging. Aging is associated with a complex network of interrelated processes classified into three categories: primary hallmarks (blue) such as genomic instability, telomere attrition, epigenetic alterations and loss of proteostasis are considered potential initiating triggers. Antagonistic hallmarks (green), including deregulated nutrient sensing, mitochondrial dysfunction and cellular senescence, are responses that can be beneficial in moderation but become harmful when exacerbated. Integrative hallmarks (red), such as stem cell exhaustion, altered intercellular communication, chronic inflammation and dysbiosis, are thought to contribute to tissue dysfunction and organismal decline. This framework provides a conceptual model to describe the multifactorial nature of aging, though causal relationships remain under investigation.

Aging is a multifaceted biological phenomenon characterized by the progressive erosion of physiological homeostasis, which culminates in functional decline across tissues and increased susceptibility to age-associated diseases and mortality^1^. A central hallmark of cellular aging is genomic instability, driven by the accumulation of DNA lesions and a concomitant reduction in the efficacy of repair mechanisms^2^. This instability is further aggravated by telomere attrition resulting from repeated cellular divisions, ultimately triggering cellular senescence or apoptosis^3^. Epigenetic alterations, including aberrant DNA methylation patterns and histone modifications, disrupt transcriptional programs and contribute to the progressive loss of cell identity and function^4^. Concurrently, the proteostasis network becomes increasingly impaired. Deficiencies in protein folding, degradation and clearance lead to the intracellular accumulation of misfolded and aggregated proteins that interfere with essential cellular processes^5^. Mitochondrial dysfunction, which is widely regarded as a hallmark of aging, is characterized by a reduced bioenergetic output and an elevated production of reactive oxygen species, thereby amplifying oxidative stress^6^. In parallel, low-grade chronic inflammation (‘inflammaging’) emerges as a systemic driver of molecular damage and tissue degeneration^7^. Aging tissues accumulate senescent cells that secrete proinflammatory factors comprising the SASP, which in turn drives further tissue dysfunction. However, recent studies have shown that senescent cells also play beneficial roles in specific physiological contexts such as tissue remodeling, embryogenesis and wound repair, indicating that their effects are highly context dependent^8^. Persistent or unresolved senescence, rather than transient responses, appears to be more closely linked to age-associated pathology. Aging also entails the dysregulation of evolutionarily conserved nutrient-sensing pathways, including the insulin–IGF-1 axis, AMPK and mTOR, which shift from cytoprotective to deleterious modes of action with age^9^. A concomitant decline in autophagy flux further compromises cellular homeostasis by impairing the clearance of damaged organelles and protein aggregates^10^. Stem cell exhaustion underlies the deterioration of regenerative capacity in aged tissues^11^, a process compounded by age-dependent alterations in the tissue microenvironment, impaired intercellular communication and chronic inflammatory signaling, all of which disrupt stem cell niches and immune competence^12^. Moreover, age-associated dysbiosis of the gut microbiota has emerged as a key regulator of host metabolism and immune tone, further exacerbating systemic decline^13^. These biological features are commonly described as the ‘hallmark of aging’, forming a conceptual framework rather than a definitive set of causal mechanisms. Although interrelated, their individual contributions to aging and disease remain areas of active investigation. Clarifying these relationships is essential for developing effective interventions aimed at extending healthspan and delaying age-related pathology^14^ (Fig. 1).

**Fig. 2:**
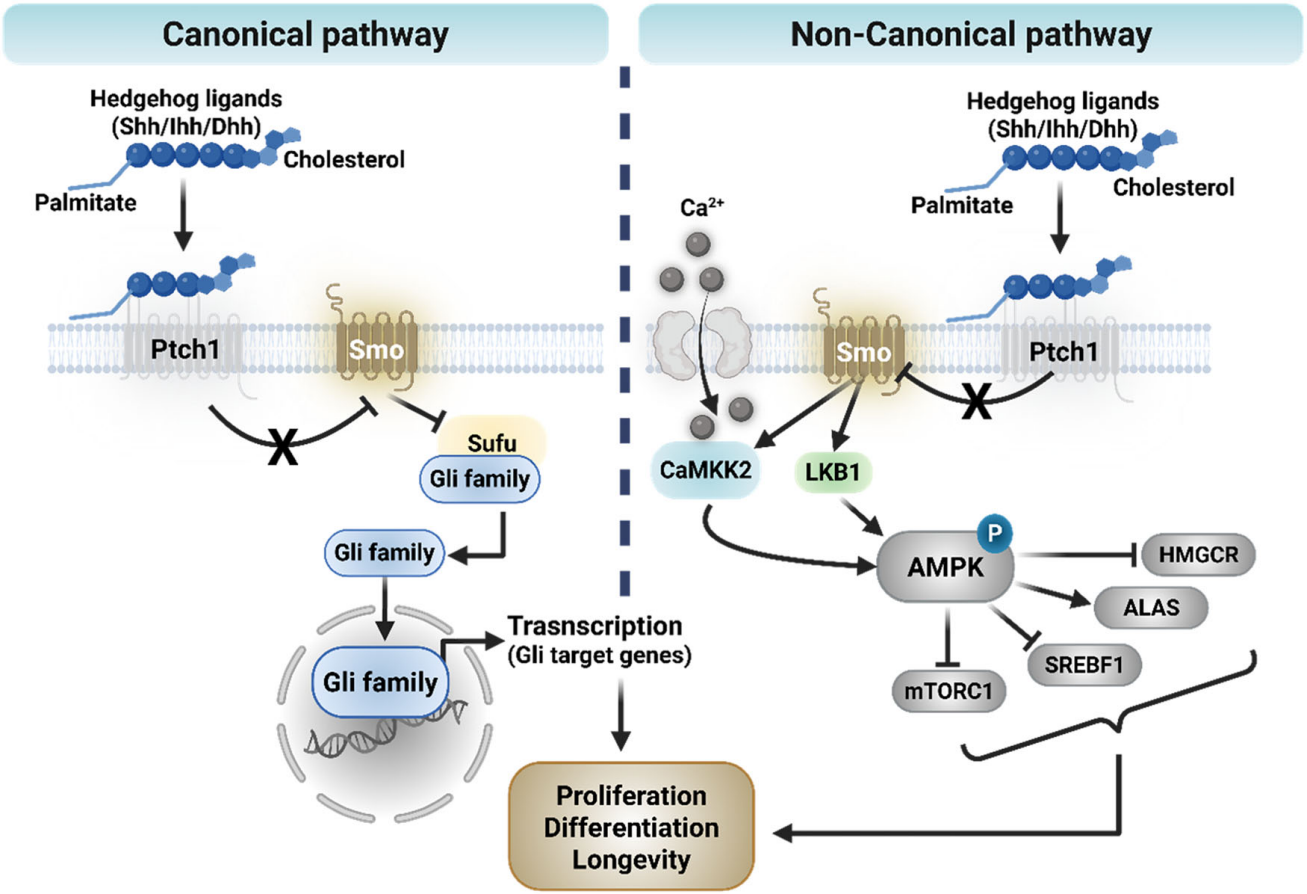
Hh signaling pathway. Left: in the canonical pathway, Hh ligand binding to Patched 1 (Ptch1) relieves its inhibition of SMO, leading to the activation of Gli transcription factors and the regulation of target gene expression. Right: in the noncanonical pathway, SMO activates downstream signaling independent of Gli, including the CaMKK2–LKB1–AMPK axis, which modulates metabolic regulators such as mTORC1, HMGCR, ALAS and SREBF1, thereby influencing cellular proliferation, differentiation and longevity.

The Hedgehog (Hh) signaling pathway is a conserved developmental signaling system that regulates key processes such as embryogenesis, tissue organization and stem cell maintenance^15^. Although first discovered in *Drosophila*, studies in vertebrates have shown that the pathway plays a central role in controlling spatial patterning during organ development^16^. In mammals, Hh signaling is initiated when one of the three secreted ligands, namely Sonic Hedgehog (Shh), Indian Hedgehog (Ihh) or Desert Hedgehog (Dhh), binds to the Patched receptor (PTCH1 or PTCH2), which inhibits the activity of Smoothened (SMO) under basal conditions^17^. Ligand binding blocks this suppression, resulting in SMO activation and the initiation of downstream intracellular signaling events that ultimately regulate transcriptional outputs^18^. The main effectors of the pathway are the GLI transcription factors (GLI1, GLI2 and GLI3). Their activity is modulated through interactions with negative regulators such as Suppressor of Fused (SUFU), as well as through posttranslational modifications. GLI proteins can function either as transcriptional activators or repressors depending on the cellular context, enabling a broad range of context-specific gene expression responses^19^ (Fig. 2). During development, Hh signaling governs cell fate specification in various organs including the neural tube, limbs and gastrointestinal tract^17^. In adult tissues, the pathway is typically inactive under homeostatic conditions but can be reactivated during tissue injury and regeneration^18^. Persistent or misregulated activation of Hh signaling has been associated with tumorigenesis in several cancer types, such as basal cell carcinoma and medulloblastoma. The inhibition of SMO with small molecules has shown clinical efficacy in several of these cases, though therapeutic resistance and alternative activation routes remain important challenges. These findings have established Hh signaling as a key regulatory system not only in development but also in tissue repair and disease. A better understanding of how the pathway is controlled in different contexts may support the development of more effective therapeutic strategies.

Although Hh signaling is well recognized for its roles in development and cancer, growing evidence indicates that it also contributes to tissue maintenance, regeneration and immune regulation during aging. These functions align closely with several hallmarks of aging, including stem cell exhaustion, mitochondrial dysfunction and chronic inflammation. In this Review, we explore the emerging roles of Hh signaling in aging biology across multiple tissues and assess its potential as a therapeutic target for promoting healthy aging.

## Hh signaling in tissue maintenance and regeneration during aging

### Brain

Aging of the central nervous system is characterized by reduced neurogenesis, increased neuroinflammation and impaired synaptic plasticity, all of which contribute to cognitive decline and increased vulnerability to neurodegenerative disorders. The Shh signaling pathway has emerged as a critical regulator of neurogenesis and neuronal survival, offering promising avenues for intervention in age-related neurological conditions. Zou et al. demonstrated in an adult mouse model of chemotherapy-induced peripheral neuropathy that Shh restrains ubiquitin-dependent degradation of synaptic proteins via the TRIM25–CXCL13 axis, mitigating neuronal and glial senescence-associated phenotypes^20^. These results support the neuroprotective capacity of Shh in aging brains through the stabilization of synaptic components. Multiple studies have highlighted the role of Shh in adult neurogenesis. Shh promotes hippocampal neurogenesis and memory formation in aged mice^21^. Similarly, Yao et al. ^22^ uncovered a dendrosomatic mode of Shh signaling in hippocampal neurons that is essential for maintaining synaptic integrity under stress conditions^22^. In the context of neurodegenerative diseases, Yao et al. ^23^ reported that Shh pathway activation enhances mitochondrial function and promotes neuronal survival in models of Parkinson’s disease^23^. These mitochondrial effects may be particularly relevant given the growing appreciation for metabolic dysfunction as a driver of neurodegeneration. Moreover, Chen et al. ^24^ emphasized emerging roles of Shh in adult neural stem cell niches, where it helps maintain progenitor pools and supports differentiation^24^. This stem cell-supportive function makes Shh an attractive target for the age-related loss of regenerative capacity in the brain.

Collectively, these studies establish Hh signaling as a key pathway in preserving cognitive function, supporting adult neurogenesis and defending against neuronal stress in the aging brain.

### Liver

The liver has a remarkable ability to regenerate after injury; however, this regenerative capacity is notably impaired during aging and chronic disease. Emerging evidence highlights the role of Hh signaling in regulating liver homeostasis, particularly by modulating hepatocyte viability, hepatic stellate cell function and progenitor cell activation. Ochoa et al. (2010) were the first to report that systemic treatment with a pharmacologic inhibitor of Hh signaling prevented adult murine liver regeneration after acute 70% (partial) hepatectomy^25^. Using a mouse model of conditional, cell-specific disruption of the Hh pathway, Michelotti et al. subsequently showed that Hh pathway activation in myofibroblasts was required to support regenerative responses in hepatocytes following partial hepatectomy^26^. These findings identified a previously unsuspected Hh-dependent paracrine axis for adult liver repair and were consistent with earlier research that had shown that other cell types involved in liver wound healing responses (for example, hepatic stellate cells^27^, cholangiocytes^28^, liver sinusoidal endothelial cells^29^ and resident and infiltrating immune cells^30^) were also capable of producing and responding to Hh ligands. Thus, converging lines of evidence support the concept that a paracrine Hh signaling network plays a major role in orchestrating the recovery of functional hepatic parenchyma after liver injury. Maeso-Diaz et al.’s (2022) more recent studies of mice with acute, conditional hepatocyte-specific disruption of Hh signaling have demonstrated that selectively disrupting Hh signaling in mature hepatocytes also suppresses liver regeneration after partial hepatectomy^31^. This latest discovery that cell-autonomous Hh activity is necessary to support mature hepatocyte proliferation complements and extends earlier evidence that Hh signaling promotes the outgrowth of liver epithelial progenitors^32^, as well as Nault’s^33^ recent discovery that the constitutive activation of Gli in hepatocytes is sufficient to cause hepatic adenomas in adulthood^33^. Thus, the cumulative data suggest that Hh may be a master regulator of adult liver homeostasis.

This concept is further supported by recent work from Chen et al. ^34^ showing that the conditional disruption of the Hh pathway in the hepatocytes of healthy young adult mice is sufficient to induce hepatocyte insulin resistance, disrupt hepatocyte lipid and cholesterol metabolism, induce redox stress, shorten telomeres and launch transcriptional programs that accelerate aging and promote hepatocyte senescence within 2 weeks^34^. Maeso-Diaz et al. (2022, 2023) showed that chronological aging impairs Hh signaling in the hepatocytes of wild type mice and found that Hh signaling-deficient hepatocytes acquire a senescence-associated secretory phenotype (SASP) that releases Hh ligands and other paracrine factors that promote hepatic stellate cells, macrophages and endothelial cells to transition into states that modulate proinflammatory and profibrogenic wound healing responses^31,35^. A recent study by Jun et al. demonstrated that reactivating Hh signaling in senescent hepatocytes mitigates these deleterious effects of the SASP, thereby restoring tissue homeostasis and preventing liver damage^36^.

The aggregate liver data illustrate the importance of context in determining the ultimate outcomes of Hh signaling. The tightly scripted activation of Hh signaling in various types of liver epithelial and stromal cell is essential for effective maintenance and recovery of functional hepatic parenchyma. However, pathway overactivation in liver stromal and immune cells promotes fibrogenesis and inflammation, and in liver epithelial cells, Hh overactivity supports neoplasia^33^. Conversely, overly suppressing the pathway in hepatocytes reduces metabolic flexibility, and the resultant loss of resiliency leads to hepatocyte senescence and the acquisition of SASPs that perpetuate maladaptive repair and, thereby, progressive liver degeneration. Dutta et al. provided comprehensive reviews of the dualistic nature of Hh signaling in liver pathophysiology^37^. Whereas persistent or uncontrolled activation may promote fibrosis and cancer, carefully regulated or transient Shh activation supports hepatocyte survival, bile duct regeneration and the expansion of progenitor cells during injury resolution. Translating these insights into therapeutic potential, Martin et al. ^38^ demonstrated that the localized delivery of Shh using peptide amphiphile nanofiber hydrogels effectively restored liver function in a radiation injury model^38^. Similarly, Hai et al. ^39^ demonstrated that *Shh* gene delivery reduced hepatocyte apoptosis and promoted tissue regeneration following radiation-induced liver injury^39^.

Together, these studies establish Hh signaling as a context-dependent but fundamentally protective and regenerative pathwaçy in the liver. The modulation of Hh activity may therefore represent a promising anti-aging therapeutic strategy for maintaining hepatic function and resilience in aged or damaged livers.

### Cardiac

Cardiac aging is characterized by a progressive decline in myocardial function, impaired regenerative capacity and increased vulnerability to ischemic injury. The Hh signaling pathway, originally discovered for its role in embryonic patterning, has been increasingly recognized as a critical regulator in maintaining cardiac homeostasis and promoting regeneration in the adult heart. Recent studies have highlighted the involvement of Hh signaling in cardioprotection and myocardial repair following injury. For instance, Zhang et al. ^40^ reviewed major signaling cascades involved in myocardial infarction and emphasized the regenerative and anti-apoptotic roles of Shh signaling in cardiac tissue remodeling and angiogenesis^40^. Shh activation post infarction enhances neovascularization and reduces myocardial fibrosis, suggesting a therapeutic potential for modulating Hh signaling in age-related cardiac dysfunction. In a more mechanistic study, Pradhan et al. (2018) demonstrated that Shh signaling directly regulates the regenerative response in mammalian cardiomyocytes^41^. Using lineage tracing and injury models, the authors showed that Shh signaling enhances the proliferation of cardiac progenitor cells and promotes tissue regeneration. Importantly, this regenerative role diminishes with age, correlating with the reduced responsiveness of aged cardiac tissue to Shh stimulation. Earlier foundational work by Liu et al. ^42^ further established that Hh signaling, in cooperation with RAS pathways, supports the anti-apoptotic survival of cardiomyocytes under stress conditions^42^. This cooperation underlies a broader cardioprotective effect, positioning Hh signaling as a candidate target for therapies aimed at reducing cardiomyocyte loss during aging or ischemic events.

Together, these findings position the Hh pathway as a key anti-aging modulator in the heart, promoting regeneration, survival and vascular integrity. Future strategies to enhance Shh signaling in aged or damaged cardiac tissue may provide novel therapeutic avenues to combat age-associated cardiovascular decline.

### Lung

The lungs are continuously exposed to environmental stressors and pathogens, resulting in significantly decreased regenerative capacity with age. Hh signaling has emerged as a key player in lung development and adult tissue homeostasis, particularly in maintaining epithelial integrity and modulating mesenchymal–epithelial interactions during injury and repair. Recent studies suggest that enhancing Hh activity may offer protective and regenerative effects in age-related lung disorders. A notable recent study by Ye et al. identified the regulatory role of Hh-interacting protein (Hhip) in orchestrating alveolar regeneration^43^. The authors demonstrated that the modulation of Hhip can fine-tune the Shh pathway, thereby facilitating alveolar epithelial repair and restoring lung architecture following injury. These findings propose a novel route for enhancing endogenous regenerative capacity through targeted Hh modulation. Further emphasizing its therapeutic potential, Wang et al. (2019) reviewed the diverse roles of Hh signaling in adult lung repair, underscoring its function in maintaining airway epithelial stem cell niches and suppressing fibrotic remodeling^44^. This is especially relevant in the context of chronic lung diseases, such as idiopathic pulmonary fibrosis, where the loss of epithelial homeostasis and aberrant repair responses accelerate functional decline. Earlier mechanistic studies also highlighted the contribution of Hh signaling to lung morphogenesis and senescence resistance. For example, Li et al. showed that the Shh-mediated regulation of Gli3 processing is essential for proper lung branching and alveolarization during development, which lays the foundation for resilient lung structure into adulthood^45^.

Collectively, these findings indicate that Hh signaling supports lung tissue maintenance and regeneration. Augmenting this pathway may present a promising strategy to combat aging-related pulmonary dysfunction and improve outcomes in chronic lung diseases.

### Bone and cartilage

Bone aging is accompanied by reduced bone formation, increased fragility and altered remodeling dynamics, predisposing the elderly to osteoporosis and osteoarthritis. Hh signaling, long recognized for its developmental role in skeletal patterning, continues to exert regulatory influence over bone homeostasis and repair in adult tissues. Several recent studies have shed light on its anti-aging potential in maintaining skeletal integrity. Notably, Zhang et al. demonstrated that Hh signaling modulates bone homeostasis by influencing the mechanical loading response in osteocytes^46^. Their work revealed that Hh activation supports the mechanosensory function of osteocytes and sustains bone mass under physiological stress, underscoring its importance in skeletal aging. Further supporting its beneficial role, Al-Azab et al. found that Ihh signaling negatively regulates senescence in bone marrow-derived mesenchymal stem cells (MSCs), promoting their osteogenic potential^47^. This finding implies that enhancing Ihh activity could delay age-related MSC dysfunction and support lifelong bone regeneration capacity. In the context of osteoarthritis, which disproportionately affects older adults, in p27-deficient mice, Wu et al. demonstrated that the Shh–Gli–Bmi1 axis enhances chondrocyte progenitor proliferation and suppresses senescence, thereby promoting bone anabolism and delaying cartilage degeneration^48^. Similarly, several studies underscore the role of Shh in stimulating osteoblast differentiation and matrix deposition in both murine and human models^49,50^.

Overall, the evidence points to a protective and regenerative function of the Hh pathway in skeletal tissues. By preserving the viability and differentiation capacity of key progenitor populations and enhancing anabolic responses to mechanical and biochemical stimuli, Hh signaling emerges as a promising target for combating bone aging and degenerative skeletal disorders.

### Hair

Skin aging involves structural and functional deterioration of the epidermis and dermis, accompanied by reduced stem cell activity and delayed wound healing. Among the signaling pathways that preserve skin integrity, Hh signaling plays a crucial role in maintaining stem cell niches, particularly within the hair follicle. Hh signaling has been shown to support regenerative programs in dermal fibroblasts and hair follicle stem cells (HFSCs). Liu et al. (2022) reported that Hh signaling reprograms fibroblasts within the hair follicle niche into a hyperactivated, regenerative state, which enhances HFSC proliferation and tissue regeneration^51^. This suggests a role for Hh in reversing stem cell quiescence and promoting renewal in aging skin. Earlier foundational work by Rittie et al. demonstrated that Shh signaling is essential for maintaining HFSC identity and cyclical regeneration in adult skin^52^. Loss of Hh activity disrupted the quiescent state of HFSCs and impaired their regenerative response, emphasizing its requirement in sustaining long-term stem cell function.

Collectively, these studies highlight the anti-aging potential of Hh signaling in skin by supporting the structural and functional maintenance of the stem cell compartment. Modulating this pathway may offer therapeutic strategies for age-related skin degeneration and hair loss.

### Adipose

Adipose tissue plays a critical role in energy storage, endocrine regulation and metabolic health. Aging disrupts adipose tissue function by altering progenitor cell differentiation and promoting ectopic fat accumulation. The Hh signaling pathway has emerged as a key regulator of adipocyte differentiation and MSC fate, with implications for mitigating age-associated metabolic dysfunction. In a study focused on stem cell modulation, Cheng et al. reviewed the use of small molecules to direct MSC fate and highlighted Hh signaling as an effective repressor of adipogenesis^53^. The activation of Hh signaling was shown to inhibit the transcriptional cascade leading to white adipocyte differentiation, thereby promoting alternative lineages such as osteogenic or chondrogenic fates. This ability to shift MSC fate away from adipogenesis may be beneficial in the context of aging, where excessive adiposity and impaired regenerative balance are common. In addition, Zhang et al. described how Hh signaling maintains skeletal integrity via the regulation of osteocyte function and noted crosstalk with adipogenic pathways in the bone marrow niche^46^. Their findings suggest that the suppression of adipogenic differentiation by Hh signaling may contribute to both bone homeostasis and healthy adipose distribution in aging organisms.

Collectively, these studies suggest that Hh signaling has a suppressive effect on adipogenesis and may support adipose tissue health by modulating stem cell fate. The targeted activation of Hh signaling could thus serve as a strategy to reduce age-related adipose dysfunction and preserve metabolic health.

### Unclassified

Although many studies have explored the role of Hh signaling within specific organs, a growing body of evidence reveals its beneficial effects in broader biological contexts that transcend organ boundaries—ranging from angiogenesis and stem cell modulation to tissue repair and anti-inflammatory effects. These findings suggest that Hh signaling may act as a systemic regulator of regenerative and anti-aging processes. One recent study by Wang et al. (2023) demonstrated that Shh signaling promotes angiogenesis through the upregulation of VEGF and improved endothelial cell migration, offering a potential strategy for ischemia-related aging disorders^54^. This pro-angiogenic effect of Hh signaling has important implications for vascular aging, wound healing and neurovascular integrity. In a stem cell-related context, Xu et al. reported that Shh signaling enhanced neural organoid development in spinal cord regeneration models, facilitating axon patterning and glial architecture^55^. These findings suggest Hh signaling may serve as a molecular cue to rejuvenate neural tissue architecture across developmental and regenerative settings. Furthermore, O’Sullivan et al. ^56^ showed that Ihh signaling expressed by TNF-activated renal epithelial cells promotes anti-inflammatory responses and tissue homeostasis under inflammatory stress^56^. This highlights the immunomodulatory potential of Hh signaling during aging and chronic kidney injury, even in the absence of canonical pathway activation. Another compelling example is the work by Feng et al., where the systemic activation of Hh signaling was shown to modulate bone marrow-derived mesenchymal stromal cells, enhancing their survival and regenerative functions under conditions of oxidative stress^57^. These results underscore the role of Hh as a supportive niche signal that maintains stem cell viability in aging and injured environments. Lastly, Cho et al. investigated the use of Shh gene therapy in a rat model of cavernosal fibrosis and found that Hh activation suppressed fibrotic remodeling and preserved erectile tissue structure^58^. This unexpected but valuable finding extends the potential application of Hh signaling in tissue-specific antifibrotic therapies.

Together, these unclassified or systemic studies reflect the wide-ranging influence of Hh signaling in supporting cellular resilience, modulating inflammation and orchestrating tissue repair processes beyond traditional organ-based models. They point toward a future where targeted manipulation of Hh signaling could serve as a multisystemic anti-aging and regenerative strategy.

## Therapeutic potential of Hh signaling for anti-aging

The therapeutic modulation of Hh signaling has garnered increasing interest as a strategy for promoting tissue repair, counteracting senescence and extending healthspan. Given its central role in developmental processes, tissue maintenance and regeneration, both the activation and inhibition of Hh signaling are being explored across a spectrum of age-associated pathologies. This section outlines emerging approaches to leverage the Hh pathway for anti-aging therapies, emphasizing its reparative, protective and stem cell-supportive capacities (Table 1).

**Table 1:** Organ-specific anti-aging effects of Hh signaling.

Organ	Year	Mechanism	Phenotype/function	References
Brain	2025	Shh prevents proteasomal degradation of synaptic proteins	Preserved neuronal structure/function	^20^
	2016	Shh promotes hippocampal neurogenesis	Enhanced memory and neurogenesis in aged mice	^21^
	2015	Dendrosomatic Shh signaling in hippocampal neurons	Maintains synaptic integrity under stress	^22^
	2017	Shh enhances mitochondrial function	Neuronal survival in Parkinson’s models	^23^
	2018	Shh maintains adult neural stem cell pools	Supports neural progenitor differentiation	^24^
Liver	2024	Shh mitigates SASP in senescent hepatocytes	Restores liver homeostasis	^36^
	2022	Hh restoration in aged hepatic stellate cells	Improved regeneration, reduced fibrosis	^31^
	2023	Regulated Shh activation	Supports survival and bile duct regeneration	^37^
	2021	Shh hydrogel delivery	Restores function in irradiated liver	^38^
	2018	Shh gene therapy	Reduced apoptosis and promoted regeneration	^39^
Cardiac	2022	Shh-mediated angiogenesis and remodeling	Reduced fibrosis and enhanced regeneration	^40^
	2018	Shh promotes cardiac progenitor proliferation	Cardiac regeneration	^41^
	2006	Shh-RAS anti-apoptotic survival signaling	Cardiomyocyte survival under stress	^42^
Lung	2025	Hhip modulates Shh signaling	Alveolar epithelial repair	^43^
	2019	Shh maintains airway stem cell niches	Suppresses fibrotic remodeling	^44^
	2004	Shh-Gli3 processing for morphogenesis	Supports alveolarization	^45^
Bone/cartilage	2022	Hh supports osteocyte mechanotransduction	Maintains bone mass	^46^
	2020	Ihh represses senescence in bone marrow-derived MSCs	Promotes osteogenic potential	^47^
	2023	Shh–Gli–Bmi1 axis in chondrocytes	Delays cartilage degeneration	^48^
	2019	Shh promotes osteoblast differentiation	Bone formation	^49^
	2010	Shh regulates matrix deposition	Supports osteogenesis	^50^
Hair	2022	Shh reprograms follicular fibroblasts	Enhances HFSC proliferation	^51^
	2009	Shh maintains HFSC quiescence	Sustains cyclical regeneration	^52^
Adipose	2019	Shh inhibits adipogenesis	Promotes osteogenic/chondrogenic lineage	^53^
	2022	Shh suppresses marrow adipogenesis	Supports bone and adipose balance	^46^
Systemic	2023	Shh induces VEGF	Angiogenesis and endothelial regeneration	^54^
	2023	Shh enhances neural organoid patterning	Spinal cord regeneration	^55^
	2022	Ihh anti-inflammatory signaling	Tissue homeostasis in kidney	^56^
	2021	Shh enhances MSC survival	Stress resilience	^57^
	2018	Shh gene therapy in fibrosis	Preserves erectile tissue	^58^

### Targeted activation to promote regeneration

Several studies have demonstrated the capacity of Hh pathway activation to rejuvenate tissue-specific regenerative responses that are otherwise diminished with age. For instance, Zou et al. showed that Shh signaling prevents the proteasomal degradation of synaptic proteins in a chemotherapy-induced neuropathy model, mitigating neurodegenerative phenotypes^20^. In the liver, Jun et al. reported that Hh activation in senescent hepatocytes ameliorates SASP-induced microenvironmental damage and restores homeostasis^36^. Beyond genetic or viral modulation, small-molecule agonists of Hh signaling have been actively explored. SMO agonists such as Smoothened agonist (SAG) and purmorphamine activate downstream GLI transcription factors by mimicking endogenous Hh ligand activity. For example, SAG has been shown to rescue cerebellar development in down syndrome mouse models and to stimulate remyelination and neurogenesis in demyelinating injury. Notably, SAG was also incorporated into a small molecule cocktail that reversed transcriptomic aging signatures in human fibroblasts without inducing pluripotency, highlighting its rejuvenative potential^22^. Similarly, purmorphamine has been used in spinal cord injury models to promote axonal regeneration and suppress glial scar formation. In vivo delivery strategies have shown translational promise. In a rat cavernous nerve injury model, Martin et al. delivered Shh via a peptide amphiphile nanofiber hydrogel to penile tissue, suppressing intrinsic and extrinsic apoptotic signaling and preserving erectile function. Recombinant Shh-N protein, although bioactive, suffers from a short half-life and poor tissue penetration, necessitating localized or sustained-release formulations. Oxysterols, such as 20(*S*)-hydroxycholesterol, are endogenous cholesterol derivatives that allosterically activate SMO and are being explored for stem cell modulation and osteogenesis. However, caution is warranted. Chronic or systemic Hh activation carries the risk of aberrant proliferation, as seen in preclinical studies where SAG induced excessive cell growth or inflammation in a dose- and sex-dependent manner. Thus, therapeutic strategies favor localized, transient or target cell-specific activation to maximize regenerative benefits while minimizing oncogenic risks.

### Senolytic and anti-SASP applications

Aging tissues accumulate senescent cells that secrete proinflammatory factors comprising the SASP, contributing to tissue dysfunction; however, transient or context-dependent senescence also plays beneficial physiological roles in tissue remodeling, development and repair. Recent evidence has expanded our understanding of senescent cells, recognizing their context-dependent physiological roles such as in wound healing and embryonic development. Nevertheless, persistent senescence contributes to chronic inflammation and fibrosis. Novel approaches have explored the use of Hh signaling to counteract SASP components. For instance, O’Sullivan et al. showed that the modulation of Hh activity in senescent hepatic stellate cells and the renal epithelium downregulated proinflammatory pathways, thus restoring regenerative balance in fibrotic environments^56^. Rather than eliminating senescent cells directly (senolysis), this strategy positions Hh signaling as a senomorphic—capable of repressing harmful secretory phenotypes while preserving cell function.

### Neuroprotective and vascular regeneration strategies

In the central nervous system, Shh has been explored as a neuroprotective agent. Beyond promoting neurogenesis, Hh signaling supports mitochondrial function, synaptic maintenance and anti-apoptotic survival in dopaminergic neurons, as shown in preclinical models of Parkinson’s disease^23^. The activation of the pathway using SMO agonists promotes dopaminergic neuron resilience under oxidative stress. Systemically, Wang et al. (2023) reported that Shh-induced VEGF expression promotes angiogenesis and endothelial regeneration in aged vasculature, suggesting potential applications for cerebrovascular and ischemic aging contexts^54^. SMO agonists such as purmorphamine have also been evaluated for promoting endothelial repair in vitro, although clinical translation remains at early stages. Collectively, these findings support the therapeutic promise of pharmacological Hh activation in aging-related degeneration. Although no Hh agonist is approved for clinical anti-aging applications, mounting preclinical data underscores their potential, provided safety and delivery challenges are addressed.

## Future directions and challenges

Although the therapeutic activation of Hh signaling holds immense promise for combating age-related tissue degeneration and restoring regenerative capacity, several key challenges must be addressed to fully harness its anti-aging potential. These include issues related to specificity, delivery, temporal control and safety, as well as the need for a deeper mechanistic understanding of the pathway’s context-dependent effects.

### Context-specificity and dose-dependence

One of the primary obstacles in clinical translation is the dual nature of Hh signaling: although it promotes tissue repair and stem cell maintenance under controlled conditions, sustained or misregulated activation can lead to pathological outcomes such as fibrosis or tumorigenesis^18,37^. The effects of Hh signaling are highly context- and dose-dependent. For example, whereas the transient activation of Shh supports liver regeneration, chronic overactivation has been linked to hepatic fibrosis^31^. Therefore, future therapeutic strategies should emphasize the precise modulation of the pathway, ideally through tissue-specific or injury-responsive systems.

### Safe and efficient delivery modalities

Current delivery strategies—including gene therapy, peptide nanofiber hydrogels and small-molecule agonists—have shown preclinical efficacy in targeting Hh signaling to damaged tissues^38,39^. However, the challenge remains to achieve spatiotemporal precision in vivo while minimizing off-target effects. Controlled-release systems, ligand-conjugated nanoparticles and synthetic promoter systems represent emerging technologies that may improve specificity and minimize systemic toxicity. Further research is needed to validate these approaches in aged or diseased models and to optimize their scalability for human application.

### Integration with other gerotherapeutic strategies

Hh signaling intersects with several other pathways that regulate aging, including mTOR, Wnt and Notch^9,14^. It remains unclear how these signaling networks interact to coordinate tissue regeneration or contribute to senescence. Future studies should aim to map pathway crosstalk at the single-cell and spatial transcriptomic levels to uncover synergistic or antagonistic relationships that could inform combination therapies. For instance, the concurrent modulation of Hh and autophagy pathways may enhance the clearance of damaged organelles while promoting stem cell renewal.

### Biomarkers and monitoring of therapeutic response

A major limitation in current preclinical studies is the lack of robust biomarkers to monitor the activation state of Hh signaling in vivo, particularly in human tissues. The development of noninvasive imaging tools, circulating transcriptional signatures (for example, GLI1/GLI2 targets) or epigenetic markers will be critical for patient stratification and therapeutic monitoring^18,19^. These markers could also help distinguish beneficial regenerative activation from pathologic overdrive.

### Aging models and translational gaps

Most studies demonstrating the regenerative or protective effects of Hh signaling have been conducted in young or acutely injured animal models. There is an urgent need to validate these findings in physiologically aged models, which more accurately represent human aging and chronic tissue decline^1,20^. Moreover, sex- and tissue-specific differences in pathway responsiveness remain poorly characterized and warrant deeper investigation to inform personalized interventions.

### Long-term safety and tumorigenic risk

Given the oncogenic potential of aberrant Hh activation in basal cell carcinoma, medulloblastoma and other cancers^15,59^, stringent safety evaluations are essential before anti-aging applications can move into clinical trials. Strategies such as pulse-activation regimens, the incorporation of safety switches or dual-targeted vectors may help mitigate the risks of sustained GLI-driven transcriptional activation.

## Conclusion

The Hh signaling pathway, long recognized for its essential role in development and tissue organization, is now emerging as a pivotal regulator of aging biology. Accumulating evidence highlights its ability to preserve tissue integrity, support stem cell function, modulate inflammation and promote regeneration across diverse organ systems. In the context of aging, the controlled activation of Hh signaling has shown promise in reversing hallmarks of senescence, restoring homeostasis and extending tissue functionality. Although this age-associated decline in Hh activity is often viewed as detrimental, it may alternatively reflect a tumor-suppressive adaptation—a programmatic response to limit proliferative signaling and reduce cancer risk in later life^60,61^. Accordingly, the therapeutic application of Hh pathway modulation demands careful calibration owing to its context-dependent effects and potential oncogenicity. Continued efforts to develop precise delivery systems, reliable biomarkers and aged-model validations will be crucial for translating this pathway into safe and effective gerotherapeutics. As we deepen our understanding of how Hh signaling interfaces with other aging-related pathways and cellular processes, it may ultimately serve as a cornerstone in the design of future anti-aging interventions aimed at prolonging healthspan and improving quality of life.
